# Special Issue: Advances in Healthcare for Neonates

**DOI:** 10.3390/children10061048

**Published:** 2023-06-12

**Authors:** Arun Prasath, Vasantha H. S. Kumar

**Affiliations:** 1 Department of Pediatrics, University at Buffalo, Buffalo, NY 14203, USA; aprasath@buffalo.edu; 2 Division of Neonatology, UT Southwestern, Dallas, TX 75390, USA; 3 Division of Neonatology, Department of Pediatrics, University of Florida College of Medicine, Jacksonville, FL 32209, USA

We are delighted to present an editorial for the Special Issue ‘*Advances in Healthcare for Neonates*’. This Special Issue is a testament to the excellent quality of the eleven articles submitted in the short span of just one year, far exceeding our expectations. We are humbled by the commitment of the scientific community to pursue neonatal research across the globe, which bodes well for improvements in quality of life as newborns grow into children and adults.

The survival of extremely preterm infants in the past few decades is closely linked to the discovery of surfactants in the early 1990s. Advances in neonatal care, including parenteral nutrition, gentle ventilation, and infection control practices, have further contributed to the increasing survival of extremely low birth weight (ELBW) infants. The extensive use of continuous positive airway pressure (CPAP) and non-invasive ventilation (NIV) techniques is essential for the close monitoring of infants on NIV to ensure appropriate clinical decision-making. One such method is standardizing the weaning process when using non-invasive ventilatory support. Nussbaum et al. attempted to standardize the weaning of NIV using the Silverman–Andersen score (SAS) [1]. The study did not find any differences among the groups, highlighting the fact that various factors, including interrater reliability, influence weaning from NIV, thereby limiting the predictive value of the SAS [1]. However, the study addresses an important knowledge gap in weaning infants on NIV off respiratory support.

Neonatal units have traditionally used chest X-ray for the diagnosis of respiratory disorders in neonates. However, more recently, lung ultrasound has emerged as a useful clinical tool at the bedside. Ismail et al. have demonstrated that imaging using lung ultrasound can not only be used as an alternative to chest X-ray, but also as a high-precision tool for diagnosing various respiratory diseases in neonates, such as respiratory distress syndrome, pneumonia, transient tachypnea of the newborn, meconium aspiration syndrome, pneumothorax, and atelectasis [2]. Incorporating point-of care-ultrasound scanning in scientific studies and training programs would certainly enhance the existing clinical applications of ultrasound, thus helping to advance the care of neonates. 

Despite advances in neonatal care leading to the increased survival of ELBW infants, premature infants are at an increased risk of adverse long-term neurodevelopmental outcomes, including cerebral palsy. Assessment of motor movements based on heart rate is a novel way of detecting abnormal pathologies that could help in earlier detection of cerebral palsy. In this Special Issue, Maeda et al., from Japan, present an algorithm to extract the movement patterns of premature neonates, as assessed through a combination of heart rate and video recordings of general movements [3]. The authors demonstrated that it is possible to use an algorithm-based approach to assess general movements using instantaneous heart rate monitoring; however, they caution that it is essential to distinguish artifacts, such as a care intervention, using a supplemental video recording [3]. Nevertheless, as fetal movements indicate fetal wellbeing, movement pattern assessment using algorithmic tools could be valuable for assessing motor and cognitive functions in premature infants after birth. 

Early diagnosis and appropriate intervention can minimize the risk of developmental delays sometimes seen in premature neonates. A randomized controlled study comparing standardized early physical therapy versus no intervention in preterm infants from 32 weeks of gestation to 2 months corrected age demonstrated no differences between the groups [4]. However, factors such as the dose, intensity of intervention, parental compliance, and the shorter duration of intervention might have contributed to an absence of difference between the groups [4]. The authors also highlight that engaging with and educating parents demonstrating poor compliance with therapy techniques for prolonged periods is essential to derive benefits [4]. 

Implementation of neuroprotective care in the neonatal intensive care unit is essential for optimal neurodevelopmental outcomes in premature neonates. Therefore, reducing pain is critical for neuroprotective care in premature infants. Dusek et al. studied the possibilities of influencing the procedural pain associated with retinopathy of prematurity (ROP) screening using oral clonidine [5]. The authors assessed the pain and vegetative scores of using oral clonidine versus standard care during routine ROP exams [5]. Although they did not demonstrate any difference between the groups, the absence of severe complications with clonidine may make it a potential candidate in future studies addressing neonatal pain [5]. 

The clinical care of neonates is the focus of this Special Issue. Traumatic lumbar puncture (LP) has been a problem confounding the diagnostic evaluation of neonates, especially in extremely low birth weight infants. In addition to ensuring the proceduralist′s technique, skills, and experience, it is also essential to use the correct size of needle when performing a procedure. In a study in this Special Issue, a smaller gauge (25G) lumbar puncture needle not only resulted in a decreased incidence of traumatic LP, but also a reduction in desaturation episodes during the procedure [6]. This study is a step in the right direction for providing neuroprotective care to these fragile infants. Future studies should address optimal positioning, non-invasive imaging techniques to facilitate easier insertion, and needle size stratification based on gestational age or birth weight in order to optimize the success of vital neonatal procedures. 

Improving the outcomes of neonates is best accomplished by preventing hospital-acquired infections and ensuring the optimal screening of newborns in the intensive care unit. The World Health Organization has described antimicrobial resistance as a serious threat to public health; hence, screening fragile infants for multidrug-resistant microbes is essential. Out born infants admitted to neonatal units in Turkey were meticulously screened using perirectal swab cultures and were found to have a 27.2% and 4.8% positive screening rate for carbapenem-resistant Enterobacterales (CRE) and vancomycin-resistant Enterococci (VRE), among the 125 referrals from the outside hospitals [7]. This result reinforces the need for antibiotic stewardship to prevent multidrug resistance, and high vigilance and attention to screening when these vulnerable neonatal patients are referred from centers in which antibiotic policies are unclear [7]. 

Next-generation sequencing (NGS) and exome and genome sequencing using targeted panel molecular genetic analysis have contributed significantly to advances in newborn care. In this issue of *Advances in Newborn Care*, Zaza et al. describe a neonate with a cleft palate and an aortic root aneurysm, with a pathogenic mutation of exon 8 of *TGFBR2* confirming a diagnosis of Loeys–Dietz syndrome [8]. Advances in molecular genetics will help better diagnose rare conditions using genetic mutations, thus contributing to earlier detection of conditions and better management of these infants. Neonates with special conditions and genetic syndromes require a higher level of care and treatment strategies, and standardized tools to enhance their recovery. Vogt et al. propose an enhanced recovery protocol for patients undergoing the Kasai procedure for biliary atresia [9]. The checklist includes, among other elements, parental education, preoperative dextrose-containing fluids, maintaining normothermia, adequate analgesia, and initiation of early feeds [9]. The checklists almost always provide a framework for clinicians to optimize outcomes in complex patients such as those requiring the Kasai procedure. 

With the increasing survival of premature infants, many patients go home with an accompanying increase in respiratory morbidities post-discharge. The widespread use of palivizumab helps to reduce re-admission rates and complications from infection with respiratory syncytial virus. The feasibility of home immunization with palivizumab without any serious adverse events is reported in this Special Issue [10]. The advantages of home immunizations include higher parental satisfaction and well-being for the whole family [10]. This study is a step towards personalized medicine within a unique population, which may help them to avoid visiting the hospital or clinic and potentially being exposed to children with other droplet infections. On the note of personalized medicine, there is an increase in the growing adult population who were born prematurely and are thus at extremely high risk of developing various comorbidities such as systemic hypertension, metabolic syndrome, reduced exercise tolerance, pulmonary hypertension, chronic obstructive pulmonary disease, and cardiac failure [11]. Holistically addressing the problems of adults born preterm will help promote cardiovascular health, wellness, and quality of life over their lifetime [11]. Despite the large number of resources invested in the survival and care of extremely premature infants, it is surprising that minimal resources are available regarding commitment to wellness as infants grow into children and adults. Vital screening programs, effective communication, targeted counseling and therapeutic interventions, and a seamless transition of care from a pediatric clinician to an adult health care provider would improve the quality and longevity of life of those born extremely preterm.
